# Dying in acute hospitals: voices of bereaved relatives

**DOI:** 10.1186/s12904-019-0464-z

**Published:** 2019-10-31

**Authors:** Diarmuid Ó Coimín, Geraldine Prizeman, Bettina Korn, Sarah Donnelly, Geralyn Hynes

**Affiliations:** 1 End-of-Life Care, Mater Misericordiae University Hospital, Quality and Patient Safety Directorate, Eccles Street, Dublin 7, Ireland; 2 Trinity Centre for Practice and Healthcare Innovation, School of Nursing and Midwifery, Trinity College Dublin, Dublin, Ireland; 3 End-of-Life Care, Hospice Friendly Hospital Programme, 1st Floor CEO Building, St. James’s Hospital, James Street, Dublin 8, Ireland; 4 Social Work, School of Social Policy, Social Work and Social Justice, University College, Dublin, Ireland; 5 Palliative Care, School of Nursing & Midwifery, Trinity College Dublin, Dublin, Ireland

**Keywords:** End-of-life care, Palliative care, Acute hospital, Quality of care, Bereaved relatives, Quantitative, VOICES, Bereavement, Dying, Mortality feedback survey

## Abstract

**Background:**

Internationally there is an increasing concern about the quality of end-of-life care (EoLC) provided in acute hospitals. More people are cared for at end of life and die in acute hospitals than in any other healthcare setting. This paper reports the views of bereaved relatives on the experience of care they and the person that died received during their last admission in two university adult acute tertiary hospitals.

**Methods:**

Relatives of patients who died were invited to participate in a post-bereavement postal survey. An adapted version of VOICES (Views of Informal Carers - Evaluation of Services) questionnaire was used. *VOICES MaJam* has 36 closed questions and four open-ended questions. Data were gathered in three waves and analysed using SPSS and NVivo. 356 respondents completed the survey (46% response rate).

**Results:**

The majority of respondents (87%: *n* = 303) rated the quality of care as outstanding, excellent or good during the last admission to hospital. The quality of care by nurses, doctors and other staff was highly rated. Overall, care needs were well met; however, findings identified areas of care which could be improved, including communication and the provision of emotional and spiritual support. In addition, relatives strongly endorsed the provision of EoLC in single occupancy rooms, the availability of family rooms on acute hospital wards and the provision of bereavement support.

**Conclusions:**

This research provides a powerful snapshot in time into what works well and what could be improved in EoLC in acute hospitals. Findings are reported under several themes, including the overall quality of care, meeting care needs, communication, the hospital environment and support for relatives. Results indicate that improvements can be made that build on existing good practice that will enhance the experience of care for dying persons and their relatives. The study adds insights in relation to relative’s priorities for EoLC in acute hospitals and can advance care providers’, policy makers’ and educationalists’ priorities for service improvement.

## Background

In western countries, the location of death has changed significantly in the last century from people dying at home, to dying in institutional settings, such as hospitals or nursing homes [1, 2]. Acute hospitals have traditionally focussed on the diagnosis, treatment and management of serious and chronic illness; however, they are increasingly the place where care is provided at end of life and the location of death [3]. In the United Kingdom, over half of deaths occur in the acute hospital setting [4, 5]. Ireland has experienced a similar trend with 43% of all deaths occurring in acute hospitals [6].

Internationally, there is an increasing concern about the quality of palliative and end-of-life care (EoLC) provided in acute hospitals. Several reports have highlighted significant deficits and poor care provided to dying patients and their families in this setting [2, 5, 7–11]. However, studies in Ireland, including the National Audit of End-of-Life Care in Hospitals in Ireland [12] and the Survey of Bereaved Relatives, VOICES MaJam, [13] highlight many areas of good practice whilst indicating improvements that could be made to enhance EoLC in acute hospitals.

Measuring the quality of EoLC provided in healthcare settings is fundamental to quality assurance and provides information to further enhance and improve patient and family care [14–16]. Information gained from the perspective and experiences of bereaved relatives can provide a better understanding of what is important in EoLC [17] and lead to improvements in the quality of healthcare provided [15]. Understanding the knowledge gaps in this area can lead to changing the message from *‘This is what we do’* to *‘This is how we do it well’* ([18] p. 10). EoLC is defined and interpreted in literature and policy internationally in various ways, from care in the last days and hours of life [19], to a broader interpretation of the care provided to people who are likely to die within 12 months, such as people with incurable and life-limiting conditions and those who die unexpectedly, including care provided to relatives [20–23]. The broader definition of EoLC care is utilised for the purposes of this paper.

Gathering data about the quality of palliative and EoLC from patients can pose issues of bias [24, 25] and raise ethical concerns [26, 27]. To overcome such issues, surveys of bereaved relatives are widely undertaken [24, 25, 28–33]. Studies have found that bereaved relatives are an adequate proxy for patient experiences of care and useful in providing critical insights and perceptions of care experience in the time leading up to the person dying, at time of death and post death bereavement care and support [25, 34]. Furthermore, relatives indicate that taking part in post-bereavement research places a low burden on them [35] and can be therapeutic [36].

The research was undertaken in two university adult acute tertiary hospitals in Ireland. In addition to the provision of acute services for their catchment area, both hospitals provide services on a national level, typical of other Model 4 tertiary hospitals in Ireland. In 2017, the two hospitals combined, provided treatment to 46,500 inpatient visits in over 1600 inpatient beds and had 112,000 day patient visits. 539,000 outpatient visits and 108,900 emergency department visits. Both hospitals have specialist palliative care teams; comprised of a palliative medicine consultant, registrar, clinical nurse specialists and social worker who work office hours. The specialist palliative care team accept referrals from all hospital specialities based on the patients having an advanced, progressive, life-limiting condition with current or anticipated complexities that cannot adequately be managed by the primary physician and team. Similar to the other 38 acute hospitals in Ireland, both hospitals are members of the Hospice Friendly Hospitals Programme, a national initiative of the Irish Hospice Foundation in partnership with the Health Services Executive (state health agency). This programme has been described as a complex sophisticated, multi-faceted advocacy programme creating positive change in the approach to dying, death and bereavement in Irish hospitals [37–39]. The programme published the Quality Standards for End-of-Life Care in Hospitals [40]. These standards, along with the appointment of end-of-life care coordinators in a number of hospitals, have been described as key drivers for quality improvement in EoLC in acute hospitals in Ireland [37–39, 41]. Both hospitals have end-of-life care committees with membership from management, administrative and clinical staff including representation from the specialist palliative care team and public interest representatives. The committee is focussed on the implementation of the Quality Standards for End-of-Life Care in Hospitals [40] and initiatives raised by staff to introduce a palliative care approach for those who would benefit from it in each hospital.

The VOICES MaJam study [13] was undertaken against a backdrop of considerable numbers of people dying in acute hospitals in Ireland [6], the limited research conducted to date on this topic, and the lack of appropriate instruments for evaluating end-of-life care from relatives’ perspective. The aim of this study was to ascertain the quality of end-of-life care in the acute hospital setting from the perspective of bereaved relatives. The study also set out to identify aspects of satisfactory EoLC and highlight areas where improvements could be made. It is within these contexts of EoLC that this study took place.

## Methods

### Background and design

This was a quantitative descriptive post-bereavement study which gathered data retrospectively, using a postal survey, from relatives or friends of patients who died in two adult acute hospitals. An adapted version of the VOICES (Views of Informal Carers – Evaluation of Services) questionnaire [42, 43] was utilised.

### Development of the VOICES MaJam questionnaire

The National Survey of Bereaved People VOICES is an established method of collecting information on the quality of care provided by the health service to a relative or friend in England [30]. Several studies have utilised an adapted version of the VOICES questionnaire indicating good reliability and validity [2, 44–47].

The VOICES survey uses a validated questionnaire and focusses on those aspects of care which are known to be indicative of the quality of care for patients nearing end of life and their families. The VOICES survey includes, hospitals (including NHS and non-NHS hospitals), hospices, care homes and the persons’ home [43]. Our aim was to ascertain the quality of care in two acute hospitals, therefore only questions related to this setting were utilised from the VOICES questionnaire. Permission from NHS England was granted, and the adapted questionnaire was named VOICES MaJam to reflect the adaptation and involvement of both hospitals. This allowed data to be collected that would ensure that the respective hospitals were meeting the principles of care outlined in key national standards, specifically the National Healthcare Charter [48], the National Standards for Safer Better Healthcare [49] and the Quality Standards for End-of-Life Care in Hospitals [40].

The VOICES MaJam questionnaire contains 29 core questions and an additional seven questions requesting personal demographic information. In addition, the four open-ended questions from the VOICES original questionnaire were included to gather descriptive data about the care experience during the patient’s last admission to hospital. These questions were:

- *What, if anything, do you feel was good about the care?*
- *What, if anything, do you feel was bad about the care?*
- *Please use the space below if there is anything more you would like to add about the care provided by the hospital to your relative/friend during their last admission.*
- *Is there is any other help or support that you would have liked to receive from the hospital since your relative’s death, please feel free to comment below*

The questionnaire was designed to gather data on the quality of EoLC which included the following areas; dignity and respect, pain and symptom management, support provided to families, the care environment, communication and decision-making. Several new questions were developed in areas such as the provision of care in single rooms at end of life, the hospital environment and bereavement support in line with national standards [40, 48, 49], previous research [12], and statutory reports [11], that identified these areas as priorities within the Irish context. Prior to undertaking the fieldwork, the questionnaire was tested for face and content validity with a panel of twelve people who had experienced bereavement.

### Sample selection

**Fig. 1:**
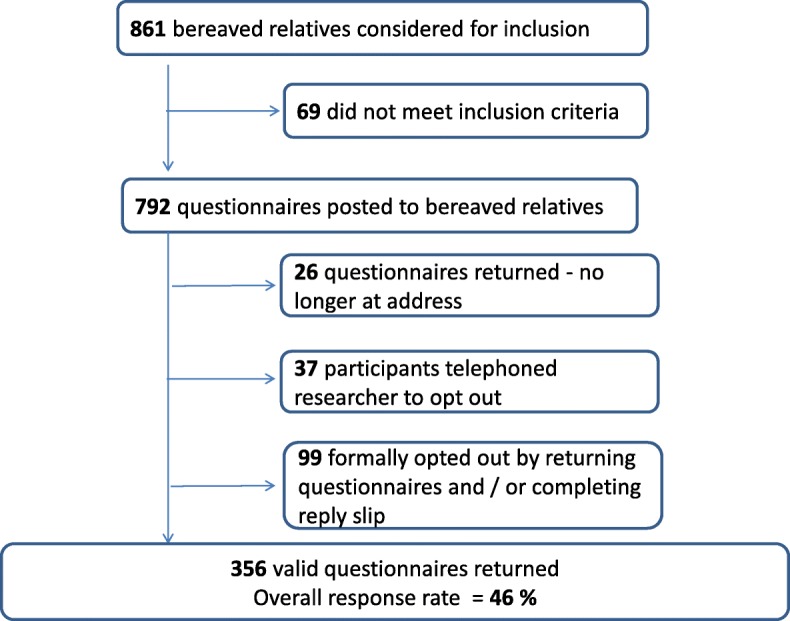
Sampling process employed

Persons recorded as the contact person in the deceased person’s healthcare record were recruited for the sample. Relatives of people who died from August 1st 2014 to January 31st 2015 were included. The sample included relatives who were bereaved no earlier than 3 months and no later than 9 months, in line with guidelines highlighted in other research [50, 51]. All deaths, including sudden and unexpected deaths were included. Demographic data collected in the questionnaire did not include the clinical area where the death occurred. Data relating to the international classification of disease (ICD) code at time of death for patients was also not captured. VOICES utilises the death certificate to recruit their sample as the Office for National Statistics conducts the survey. This was not possible with our study. The exclusion criteria for this study included the following; patients aged less than 18 years of age; patients who did not die in the hospitals; and, relatives with a missing or incomplete address. The combined number of relatives (sampling frame) was 792 (Hospital A: *n* = 385: Hospital B: *n* = 407) (Fig. 1).

### Data collection

**Fig. 2:**
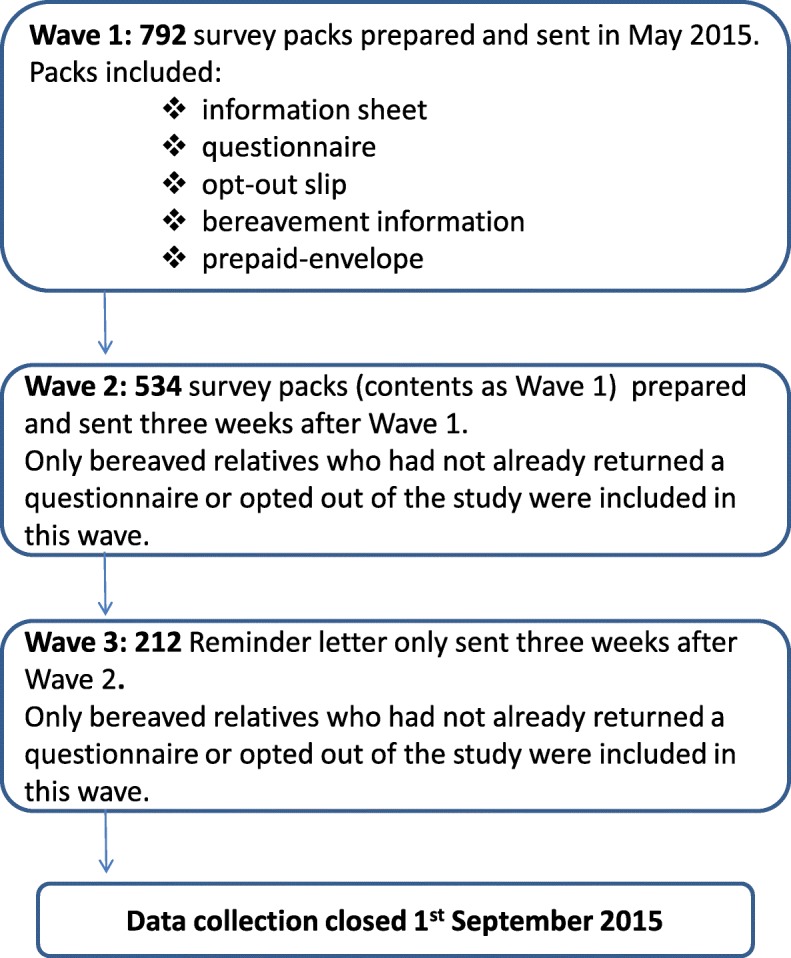
Data collection process employed

Data were collected in three waves between May and September 2015 (Fig. 2). Experience from previous VOICES surveys suggests that two reminders are optimal and are associated with a considerable increase in response thereafter [42, 43]. Respondents were provided with a study information sheet along with an opt-out reply slip, offering them the opportunity to withdraw from the study. Return of the questionnaire was viewed as consent to participate. Information on bereavement supports was included with the survey pack. Relatives were also provided with contact details of the principal investigator in each hospital if they needed to raise any queries or concerns about the study. Wave 2 mailing included the complete survey pack while Wave 3 consisted of a reminder letter only.

A total of 356 valid questionnaires were returned (Hospital A; *n* = 167: Hospital B; *n* = 189) giving an overall response rate of 46% (Fig. 2). This is relatively high for a postal survey of bereaved relatives’ [32, 45, 52, 53] but comparable with the VOICES survey in England [30] and the National Audit of End-Life-Care in Hospitals [12].

### Data analysis

Quantitative data were inputted into MS Excel and converted to the statistical package IBM SPSS statistics v. 22 [54] for analysis. Frequency and distribution tables were generated for all data. Chi-square tests of significance were carried out to examine the association between several variables. The literature suggests that the key domains of end-of-life care include management of pain relief, other symptom relief, spiritual support, emotional support, personal and nursing needs. Associations with quality of care and these domains were examined. In addition, associations between quality of care and length of stay in hospital and dying in a single occupancy room were examined to see if these had a significant impact on quality of care. A significance level of *p* ≤ 0.05 was used for all analyses.

Qualitative data were transcribed verbatim and were coded and analysed using NVivo 10 [55]. Due to the large amount of qualitative data derived from the replies of 286 relatives, a coding frame was developed based on the principles and standards of care outlined in the National Healthcare Charter [48]; the National Standards for Safer Better Healthcare [49] and the Quality Standards for End-of-Life Care in Hospitals [40]. Data were analysed thematically using a template analysis framework approach [56]. Five key themes emerged: communication, meeting care needs, hospital environment, dignity and respect and support for relatives. Inter-rater reliability tests were conducted by two independent researchers, indicating a kappa score of 0.62. An EoLC Coordinator from each hospital and experienced researchers from their respective academic partners provided a unique balance of practice and academic expertise in all analyses.

This paper reports on the quantitative findings with some selected qualitative comments that are representative of many similar comments made by bereaved relatives and are included here to provide context. A more comprehensive report of the qualitative findings is published elsewhere [57].

## Results

### Demographic details of survey respondents and deceased patients

Three quarters of respondents were female (74.2%: *n* = 259) and over four in ten (42.9%: *n* = 150) were over the age of 60 years. Children of the deceased comprised the largest group (41.1%: *n* = 144), followed by husband (22.0%: *n* = 77) or wife (12.9%: *n* = 45), including civil partner and partner. One in ten (10.9%: *n* = 38) were siblings of the deceased (Table 1).

**Table 1:** Demographics of respondents

Gender (*N* = 349)	% (N)
Male	25.8% (90)
Female	74.2% (259)
Age (*N* = 350)	% (N)
18–29 years	0.9% (3)
30–39 year	5.7% (20)
40–49 years	18.9% (66)
50–59 years	31.7% (111)
60–69 years	16.6% (58)
70–79 years	16.8% (59)
80 + years	9.4% (33)
Relationship to the deceased (*N* = 350)	% (N)
Children	41.1% (144)
Husband	22.0% (77)
Wife	12.9% (45)
Siblings	10.9% (38)
Other relative or friend	8.0% (28)
Parent	5.1% (18)

Six in ten (57.3%: *n* = 199) people that died were male and half (50.1%: *n* = 178) were over 80 years of age, with a further 35.5% (*n* = 126) between the age of 60 and 80, many with multiple co-morbidities. The largest proportion (28.9%: *n* = 99) spent between 2 days and 2 weeks in hospital on their last admission. All other patients’ length of stay was almost evenly spread between up to 48 h, 2–4 weeks, 1–2 months and longer than 2 months (Table 2).

**Table 2:** Demographics of the deceased

Gender (*N* = 347)	% (N)
Male	57.3% (199)
Female	42.7% (148)
Age (*N* = 355)	% (N)
18–29 years	1.7% (6)
30–39 years	3.2% (11)
40–49 years	3.9% (14)
50–59 years	5.6% (20)
60–69 years	10.7% (38)
70–79 years	24.8% (88)
80 + years	50.1% (178)
Length of stay in hospital during last admission (*N* = 342)	% (N)
< 48 h	18.1% (62)
2 days – 2 weeks	28.9% (99)
2–4 weeks	17.3% (59)
1–2 months	16.4% (56)
> 2 months	19.3% (66)

### Overall quality of care

The majority of respondents (86.8%: *n* = 303) rated the quality of care as ‘*outstanding*’, ‘*excellent’* or ‘*good*’. One in eight (11.8%: *n* = 41) respondents rated care as ‘*fair*’ or ‘*poor*’ (Table 3). The quality of care rated as ‘*exceptional*’ or ‘*excellent*’ was highest where care was provided by nurses (79.6%: *n* = 317) and then by doctors (71.5%: *n* = 299) followed by other staff (68.7%: *n* = 226) as shown in Table 3. One in 10 respondents rated care provided by hospital doctors (8.9%: *n* = 30) as being ‘*fair*’ or ‘*poor*’ whilst 5.1% (*n* = 18) rated care provided by nurses as ‘*fair*’ or ‘*poor*’.

**Table 3:** Ratings of quality of care

Overall quality of care - last admission	Outstanding	Excellent	Good	Fair	Poor	Don’t Know	Total
	33.5% (117)	36.4% (127)	16.9% (59)	7.2% (25)	4.6% (16)	1.4% (5)	349
Quality of care - staff group	Exceptional	Excellent	Good	Fair	Poor	Don’t Know	Total
Doctors	38.3% (129)	33.2% (112)	17.2% (58)	6.2% (21)	2.7% (9)	2.4% (8)	337
Nurses	45.0% (152)	34.6% (117)	14.2% (48)	4.4% (15)	0.9% (3)	0.9% (3)	338
Other staff	35.0% (115)	33.7% (111)	18.2% (60)	5.2% (17)	2.1% (7)	5.8% (19)	329

Tests for significance indicated that there was an association between overall quality of care and pain relief, other symptom relief, spiritual support, emotional support and support to stay where he/she wanted, physical comfort needs being met and having adequate privacy (Table 4). There was no significant relationship between quality of care and length of stay in hospital or dying in a single occupancy room.

**Table 4:** Test of significance results (chi square test for independence)

Question	N	*P* value^a^	Phi value^b^
During last 2 days, overall level of support in following areas:			
How well pain was relieved	247	*P* = .000	.28
Had enough help to meet personal care	295	*P* = .000	.48
Had enough help with nursing care	308	*P* = .000	.55
Had adequate privacy	313	*P* = .000	.40
During last admission			
Relief of pain	279	*P* = .000	.57
Relief of other symptoms	280	*P* = .000	.68
Provision of spiritual support	249	*P* = .000	.45
Provision of emotional support	255	*P* = .000	.58
Provision of support to stay where patient wanted	218	*P* = .000	.65

^a^ Not applicable or don’t know were not included in the analysis

^b^ Strength of association – with .21 indicating a medium effect and .35 a large effect

### Meeting care needs

There was variation in each domain of care as outlined in Table 5.

**Table 5:** Ratings of symptom management and support during last admission

	Excellent	Good	Fair	Poor	N/A	Don’t Know	Total
Relief of pain- last admission	48.7% (163)	25.1% (84)	6.6% (22)	4.5% (15)	11.3% (38)	3.8% (13)	335
Relief of other symptoms – last admission	42.8% (137)	29.7% (95)	10.0% (32)	6.6% (21)	6.3% (20)	4.6% (15)	320
Spiritual support – last admission	39.4% (129)	21.1% (69)	8.6% (28)	8.9% (29)	10.4% (34)	11.6% (38)	327
Emotional support – last admission	36.1% (119)	20.3% (67)	12.7% (42)	10.0% (33)	7.9% (26)	13.0% (43)	330
			Strongly Agree/Agree	Neither Agree nor Disagree	Disagree/Strongly Disagree	Don’t Know or N/A	Total
There was enough help to meet physical comfort needs in last 2 days			73.3% (250)	6.7% (23)	8.2% (28)	11.8% (40)	341

Bereaved relatives were asked to rate how staff responded to the persons pain during the last admission to hospital. The majority (87%) rated the management of pain as ‘good’ or *‘excellent’* in those who experienced pain during the last admission to hospital. Three quarters (75.6%) of those experiencing pain in the last 2 days of life, indicated that pain was *‘completely’* relieved *‘most’* or *‘all of the time’*. Many respondents commented the importance of pain relief on:

*“She was made comfortable with pain relief as she was in terrible pain and this obviously gave us some comfort at a terrible time.”*

However, a number of relatives also commented that pain was poorly managed outside of regular working hours and suggested that specialist palliative care team members should be available out of hours and at weekends to support the management of pain:

“*Unfortunately there was no palliative care personnel in the hospital on a sat/sun and I really felt dad was in quite a lot of pain on those days. Emergency & locum Doctors attended dad but they seemed to lack experience of palliative care & were not inclined to give pain medication. On the Monday, the palliative care team returned to the hospital and dad’s pain relief medication was corrected and he received adequate pain relief. Dad passed away on the Tuesday morning.”*

A large number of respondents (72.5%: *n* = 232) indicated that other symptoms were managed at the level of *‘good’* or *‘excellent’*. A further 6.6% (*n* = 21) stated other symptoms were managed poorly. In at least three out of four cases (73.4%: *n* = 250) the patient’s physical comfort needs were met in the last 2 days of life. Despite this, nearly one in 10 respondents (8.2%: *n* = 28) indicated that the patient’s physical comfort needs were not adequately met (Table 5).

Several respondents commented on the provision of personal care:

“*The care of my mother … was outstanding; my mother was treated with love care and respect until the minute she died … They were so kind to her, doing small things such as putting curling tongs in her hair. This was on top of her usual personal hygiene and nursing care any concerns raised was immediately attended to … ”*

Several relatives perceived staff shortages and difficult working conditions as impacting on the provision of patient care:

*“We felt the nurses were very overworked and just didn’t have the time to give my father the attention he needed and he was reluctant to ask/bother them.”*

The categories of emotional and spiritual support received respondents’ lowest ranking (Table 5). Over half (56.4%: *n* = 186) stated that the level of emotional support was *‘excellent’* or *‘good’*. One in ten respondents (10%: *n* = 33) indicated that emotional needs were poorly met. Several commented on their relatives’ experience of emotional support:

*“ … he was not offered to speak with anyone regarding his diagnosis … His needs or wishes were not obviously discussed with him as he wasn’t offered any spiritual/counselling support following his diagnosis. This I know would have been important to him. I do not think he suffered physical pain in the last week of his life, but I do know he suffered emotionally which is every bit as bad and it shouldn’t be.”*

*“I felt that the care was good because they helped him physically and emotionally.”*

Six in 10 respondents (60.5%: *n* = 198) indicated that the spiritual support provided was *‘excellent’* or *‘good’* with one in 11 (8.9%: *n* = 29) indicating it was *‘poor’*.

### Communication

Awareness about the likelihood of dying and the quality of communication with the dying person were explored. Almost one fifth (18.4%: *n* = 64) of respondents believed their relative was aware they were likely to die and over one quarter (27.0%: *n* = 94) saw it as probable. One fifth (19.8%: *n* = 69) indicated their belief that their relative did not anticipate they were going to die, while one quarter (24.7%: *n* = 86) definitely did not expect it. One in 10 (10.1%: *n* = 35) were unsure about this (Table 6).

**Table 6:** Awareness of and communication about dying

Did your relative know he/she was likely to die?		News of likelihood to die told in a caring and sensitive way	
Yes, certainly	18.4% (64)	Yes, definitely	26.4% (58)
Yes, probably	27.0% (94)	Yes, to some extent	13.6% (30)
Probably not	19.8% (69)	No, not at all	2.7% (6)
No, definitely not	24.7% (86)	Patient did not know was going to die	18.6% (41)
Unsure	10.1% (35)	Not told relative was going to die	25.5% (56)
		Don’t know	13.2% (29)
Total	348		220

Relatives spoke of their experience of hospital staff not recognising or failing to acknowledge and communicate the person was dying. In some instances, this led to the continuation of perceived unnecessary and burdensome interventions:

*“No one knows more than family, if close. We have comparisons so we can tell when things are changing. We knew the end was close. Not once could we communicate properly with staff on this … The day mum died, I was meeting with my sister in the café to work out when to tell my brother to come back from (name of country)... Last time I saw mum, the physio was testing her walking, so I left her early. It would have been better if the physio accepted her saying No and left us to spend a little more time, just chatting and relaxing in the short time before she died.”* [13]

*“On-going tests when my mother wasn’t strong enough for them and when it was clear she was dying.”*

When respondents were asked if the news of their relatives death was conveyed in a sensitive way, over a quarter (26.4%: *n* = 58) of responding relatives (*n* = 220) answered ‘*Yes, definitely*’. A further one in seven (13.6%: *n* = 30) responded ‘*Yes, to some extent*’, while 25.5% (*n* = 56) reported that no one told their relative that they were going to die (Table 6). Respondents shared contrasting experiences of how ‘bad news’ was delivered:

*“The Doctor’s and nursing staff were very sensitive when telling us the difficult news that my mother was going to die.”*

“*Initial diagnosis of [a] fatal condition was delivered in a direct almost brutal fashion by a nurse. This may not have been the intended mode of delivery but this is what happened.”* [13]

### Hospital environment

Seven in 10 respondents (68.9%: *n* = 241) reported that their relative died in a single occupancy room (Table 7). Many respondents, whose relative was cared for and died in a multiple occupancy room, stressed the importance of care in a single occupancy room in the days before the person died:

*“We were really hoping that we could have a private room. 2 hours before mam died, we moved into a 2-bedded room. It was better than being in the 6-bedded ward but still far from ideal. Not only for us, but for the poor woman who mam had to share with. I was grateful that mam died at midnight and the lady was asleep and the place was quiet and mam had a most beautiful death. … I think it should be a priority that there is a private room for patients & family to go to die. EVERYONE DESERVES THAT*.”

*It would have been less distressing for all if he had his own room earlier. We were trying to keep him calm and other people on the ward were not that sick. I could never complain about this, his care as it was, was 100% excellent.”*

Respondents commented on both the significance of a single occupancy room at end of life, and the impact on their dying relative and the family when a single room was not available:

*“They allowed us stay with our mother, there was no single room available but the staff went over and beyond to get us a single room for the last two days of her life.”*

*“I cannot speak highly enough about the care the nursing staff gave to my relative. Unfortunately, the lack of availability of a single room was an issue. I was with my relative when she died as I stayed all night; she passed away early in the morning. My family (5 siblings) could not all stay and were not with my mum when she died. We were fortunate that she was sharing with a lovely lady who was VERY understanding of the constant visiting.”*

*“ … when she was moved to a single, private room, there was unrestricted visiting and overnight stays were allowed. This was very helpful as she died … while we were still present.”*

*“I feel that a private room should have been offered as it was felt that we couldn’t talk loudly and share experiences, a lot of final speeches were whispered which I felt took away from the final goodbye.”*

Over two thirds of respondents (69.1%: *n* = 235) agreed there was enough privacy, however, almost one in six (17.4%: *n* = 59) indicated that there was not enough privacy.

**Table 7:** Hospital environment and support for relatives

Care in a single room at time of death	Yes	No	Not sure	Total
	68.9% (241)	26.3% (92)	4.8% (17)	350
Adequate privacy in last 2 days of life	Yes	No	N/A or Don’t Know	Total
	69.1% (235)	17.4% (59)	13.5% (46)	340
Availability of family room	Yes, found helpful	No/Don’t Know	Did not receive	Total
	71.1% (219)	4.9% (15)	24.0% (74)	308
Support for relatives at time of death	Yes, definitely	Yes, to some extent	No/ Don’t Know	Total
	69.0% (240)	24.1% (84)	6.9% (24)	348
Sensitive care after death	Yes	Yes	No/ Don’t Know	Total
	94.6% (331)	4.0% (14)	1.4% (5)	350

Another aspect of the hospital environment explored was access to or availability of a family room on the ward. One in four respondents (24.0%: *n* = 74) did not have access to a family room. Almost all (98.6%: *n* = 219) of those who had access to a family room found it helpful. Many commented on the importance of having a family room on acute hospital wards and the impact it had on their experience of care and privacy:

*“We as a family never had a family room to talk to mam in private, everyone can hear your business in wards. Even on her last day when we were advised to come in. We had nowhere to make a cup of tea or sit in private we had to use a storage room, which just adds to your distress.*”

The provision of dedicated family rooms that are warm and welcoming spaces, offering comfortable seating, free tea or coffee making facilities and a sofa bed to allow a family member stay overnight were identified by relatives as important resources. In the absence of family rooms, respondents reported sitting in open plan waiting areas and discussing confidential information including being given ‘*bad news in public spaces and corridors’*.

### Support for relatives

More than two thirds of respondents (69.0%: *n* = 240) said that they had definitely been given enough support at the time of the death. A further quarter (24.1%: *n* = 84) said that they had to some extent (Table 7).

The majority (94.6%: *n* = 331) of respondents indicated they were cared for sensitively after their relative died. A minority (4.0%: *n* = 14) indicated they were not. One relative for example, commented on the support received at this time:

*“Quick access to my mother’s body in a private room. Quick arrival of a priest. Staff checking on me regularly yet giving privacy to grieve. Tea, sandwiches for family when they all arrived. No rush to leave the room, all at my pace.”*

However, others had a different experience and would have liked more information about what to do at the time of death:

*“More practical support the day of his death no one seemed to know what happened following the death e.g. arrangements for our undertaker to remove the body from the morgue etc. considering we had such a journey, staff were not helpful.”*

One in six respondents (16.5%: *n* = 57) had spoken with someone from the hospital about their feelings around their relative’s illness and death and found this helpful; 28.6% (*n* = 99) did not but would have liked to and a further 53.5% (*n* = 185) did not wish to speak with anyone about their feelings. One per cent of respondents reported having spoken with someone and reported finding this unhelpful. Social workers, doctors and nurses were the source of contact for the majority of respondents (Table 8). Relatives commented on the support they received or required at this time:

*“We deeply appreciate receiving a letter from the staff expressing their sympathy. Thank you.”*

*“We would have liked to receive a bereavement booklet and information on what services were available to us.”*

**Table 8:** Spoke to someone about death of relative

Spoke to someone about feelings around death	Yes, found it helpful	Yes, found it unhelpful	No, did not, but would have liked to	Did not wish to speak with anyone	Total
	16.5% (57)	1.4% (5)	28.6% (99)	53.5% (185)	346
Professional spoken to	Doctor	Nurse	Social Worker / Bereavement Counsellor	Chaplain	Don’t Know/Other
	28.2% (29)	24.3% (25)	29.1% (30)	11.7% (12)	6.7% (7)

## Discussion

This study is the largest survey of bereaved relatives, in an acute hospital setting to be conducted in Ireland to date and from that perspective, findings bring previously unknown information into the public domain. Our study findings add to the international picture regarding end-of-life care in acute hospitals and will enable hospitals to understand important elements of end-of-life care from the perspective of bereaved relatives and to identify priorities for service improvement.

This research found that several aspects of care influenced the provision of EoLC for people in an adult acute hospital setting in Ireland, including quality of care, care needs being met, quality of communication, the hospital environment and support provided for relatives. While other studies using adapted versions of VOICES focussed on specific illnesses [2] and research focus [45, 47], similar themes emerged in this research. Findings from other published research [17, 58] on end-of-life care in hospital settings are also reflected here.

### Quality of care

This study was conducted to establish the quality of EoLC provided in two adult acute hospitals. It is encouraging to find that the results indicate that the quality of care at end of life was generally considered to be high by respondents. This reflects well on the quality of care in both hospitals and compares favourably with research conducted on the quality of EoLC internationally [9, 30, 59, 60]. However, bereaved relatives also indicated elements of care which could be improved. Respondents’ narrative comments provided significant insights into the diversity of care experiences and offer important suggestions for improvements that should be considered as part of the provision of EoLC in acute hospitals. Similar findings have emerged from the use of the VOICES tool [2]. This study highlights that the quality of EoLC during the last admission to hospital is multifaceted involving many factors including, but not limited to, the provision of holistic care, management of symptoms, good communication and the hospital environment.

### Meeting care needs

Quality care at end of life follows a palliative care approach which addresses physical symptoms, social, emotional and spiritual needs [61]. The assessment and management of physical pain and symptoms other than pain is a major focus of EoLC [32, 62]. The dying person’s physical problems must be anticipated and proactively addressed in order to provide comfort and maximise quality of life.

Studies [12, 63] have found that the relief of pain and symptoms other than pain were well managed by hospital staff which was reflected in this study. However, our findings contrast with other studies [50, 64] where pain was considered to be poorly managed. Where respondents in this study suggested improvements relating to poor symptom management, these centred on the lack of access to specialist palliative care expertise at weekends and out of hours. These findings mirror international findings on EoLC [8] and reports of the underfunding of specialist palliative care services in England [65, 66] and Ireland [67, 68]. The provision of face-to-face palliative care services in acute hospitals from at least 9 am to 5 pm Monday to Sunday has been recommended [69, 70]. The importance of managing pain and other symptoms was indicated by relatives when they spoke of the upset they experienced where they were not managed and the impact it had on the dying person, also noted by Dunne and Sullivan [71].

The provision of emotional and spiritual support and meeting the needs, wishes and preferences the person who is dying in these domains of care is fundamental to good EoLC and is recognised in many countries in policy [21, 60], professional standards and competencies [20, 72], and in research studies [73–75]. As in other studies [24, 59, 76], emotional support was one domain of care where relatives indicated needs were not fully met. Enhancing the provision of emotional and psychological support for the dying person and their family was required as part of EoLC in hospitals has also been identified in research [15]. Living with a life-limiting illness and awareness of the imminence of one’s own death may heighten concerns about issues related to quality of life, uncertainty about the future and death. The role of medical staff in the provision of emotional support at end of life has also been highlighted [77]. According to Mistry et al. [16] EoLC should include a holistic perspective of care reporting that *“being free of emotional and spiritual burden, including the fear of dying, was considered critical in ensuring the patient’s remaining days are mentally ‘pain free’”* [16: 3].

Several studies have considered the barriers to the provision of emotional support. These included low participation of acute hospital staff in further education on the topic [78], distancing strategies to ensure their own emotional wellbeing [78], busyness [63] and staff shortages [15].

Ensuring spiritual care and support needs are met is a quality marker of good EoLC [21, 40, 79, 80]. Provision of spiritual care that responds to the needs and preferences of the person who is dying and their relatives are core elements of holistic palliative care [61]. However, in this study, relatives indicated there was limited support of the persons’ spiritual care needs which has also been found in other studies [24, 33]. There is evidence that the provision of spiritual care, for those who are seriously ill, is somewhat neglected in acute hospitals [9, 30, 81] and it has been recommended that physicians should receive adequate training in evaluating spiritual needs [82]. Research studies report that patients valued nursing staff meeting spiritual care needs, finding it a source of comfort and meaning [83] and noted the importance of spirituality in coping with a terminal illness [82, 84–86]. Many healthcare facilities, including acute hospitals have pastoral care teams, or a hospital based chaplain, who specialise in attending to the spiritual needs of patients and their family members. However, the provision of spiritual support is also a core element and competency of all healthcare staff [86, 87]. While addressing patients’ spiritual needs is key to good EoLC, there is limited clinical guidance on how clinicians might best meet these needs [80]. Koenig [86] suggests that nursing staff or social workers should conduct two-minute spiritual “screening” evaluation of all patients and when spiritual needs are identified, the health professional would then make a referral to pastoral care services. However, barriers to meeting patient’s spiritual care needs have previously been identified as a lack of confidence by healthcare staff [81] and discomfort over discussing such issues [86].

Care of people who are dying requires attention to their personal care, comfort and support needs. Whilst the majority of respondents in this study reported the dying person received adequate care and support, some relatives reported that there was inadequate support at times, specifically with personal care as described elsewhere [8, 28, 45, 88]. Respondents reported that inadequate care was a systems related issue and cited perceived staff shortages as the main reason for unmet personal care needs.

The perceived lack of support with personal care and the unmet needs associated with the provision of emotional and spiritual support are areas for further improvement to enhance the EoLC experience.

### Communication

Fundamental to all aspects of healthcare and particularly to good EoLC, is timely, sensitive and clear communication with the person who is seriously ill and their family members [89]. As reported in other research [24, 45], the results of this study indicate that staff need to be more proactive and sensitive in their communication to ensure clarity and more open discussions about prognosis and the possibility of dying so that wishes and preferences are met. Good communication centres on respecting patients’ dignity and privacy whilst ensuring their wishes and needs are heard and understood. This is of critical importance when the discussion with the patient and family is about dying. It is also recognised that there can be considerable uncertainty in identifying when someone is dying and at times changes can happen suddenly and unexpectedly. Open communication on issues related to EoLC is crucial so that the person is fully enabled to participate in making informed decisions about care at end of life. In addition, studies [90, 91] have shown that timely advance care planning (ACP) has a positive effect on patients and their families, including a reduced burden by surrogate decision makers. Through skilled and timely engagement in ACP, healthcare professionals can contribute to families’ better assessment of the quality of dying and death [92, 93]. Lack of discussion about EoLC may lead to anxiety about dying [92] with plans left incomplete and conversations not had. Other studies [24, 59, 94] have reported on bereaved relatives’ expectation that they should be better prepared by staff for the person’s death including indicating timeframes associated with prognosis [95]. Poor communication, particularly around the time leading up to the person’s death, is well documented [24, 59, 94, 96] and is an area for further improvement [28]. Competence to communicate with patients can be enhanced by training modules [15] and courses where healthcare staff learn how to conduct difficult conversations [97].

### Hospital environment

Several aspects of the hospital’s physical environment has been identified by patients, families and healthcare staff as being important in the provision of good EoLC, including the levels of privacy, care in a single occupancy room, hygiene, atmosphere and noise levels and family facilities [98–104]. Several researchers [100, 105, 106] have indicated that care for the dying person in multiple occupancy rooms was deemed inappropriate due to the noise level and the busy atmosphere. McKeown et al. [98] found that care outcomes were perceived as better when care was provided in a single occupancy room. In addition, international experts [69, 70] recommend the availability of single rooms in the provision of palliative care. In our study, the majority of respondents received care in a single occupancy room and the majority were very satisfied with their care. However, where care was provided in multiple occupancy rooms, some respondents described such rooms as being inadequate and intrusive on the experience of the person dying and their family members and advocated the importance of care being provided in a single occupancy room. It was evident in the free text comments, that without access to a single room, relatives were unable to spend treasured time together to say their goodbyes in a relaxed comfortable and private environment which mirrors the results of studies conducted by Dunne and Sullivan [71] and Stajduhar [28].

Families spend long days and many hours with relatives who are seriously unwell or dying in acute hospitals. Similar to other studies [71, 99, 106], some relatives in this study, through free text comments, advocated the importance of having appropriate family facilities on acute hospital wards, providing privacy to meet with their family members and healthcare staff. The importance of enhancing the environment to promote dignity, privacy and therefore improve the care experience for patients and their relatives through the development of family rooms is reported elsewhere [41, 107–111]. While statistical analysis indicated no significant relationship between quality of care and death in a single occupancy room, factors including the provision of EoLC in a single room and access to family friendly facilities were reported by respondents in our study as helpful resources at this time.

### Support for relatives

International research [112, 113] in the areas of oncology and palliative care has found that staff provide bereavement support to varying degrees as part of their routine practice. Bereavement support is a core function of palliative care, with Small et al. ([114] p. 1) suggesting that there is a need for *“continued support for vulnerable carers after the death”.* However, other research [115] has found that bereavement follow-up tends to be less frequent in acute hospitals. Harrop et al. [116] report relative’s difficulties in accessing support, highlighting the absence of available services and lack of information. The barriers to the provision of a post-death bereavement support have been explored in research and are predominantly related to staffing and funding [115]. Many relatives reported in this study their preference that hospital staff make contact with them, following the death of their family member which has been reported in other research [33, 117]. Furthermore, other forms of support, such as a bereavement letter with bereavement support information, and the provision of bereavement support evenings by hospital staff were viewed favourably by respondents. Studies [118, 119] have shown that organised bereavement support evenings can be a form of comfort and have a positive impact on relatives’ grieving process, reducing levels of anxiety and depression. Bereavement services held in hospitals can act as ‘endings’ and are seen as an important component of care in the acute hospital setting [120].

### Strengths and limitations of this research

To date, this is the largest survey of bereaved relatives conducted in two acute hospitals in Ireland. Findings from this study contribute to our understanding and increase our knowledge of what is important to people at end of life. Bereaved relatives describe what good EoLC should look like and highlight areas where care can be improved in acute hospitals. The findings reflect those in similar studies, therefore, adding to and strengthening international research in this specific area. Utilising an adapted version of the VOICES questionnaire, including open-ended questions, allowed for insights to be gained into how care could be improved at end of life.

We acknowledge that the study is limited somewhat by representing the views of bereaved relatives who choose to respond. We do not have demographic information on relatives who did not respond for comparison. In addition, the study does not explore the views of patients directly. While the reliability of proxy reporting has been questioned by some [121–123], others express confidence about its reasonable validity and correspondence with patient’s views [124–126]. Study respondents were in the main from a specific region in Ireland, and therefore may not be representative of bereaved relatives countrywide. Research challenges relate to the fact that the research was carried out from an ‘insider’ position thus raising issues of potential bias however; it could also be argued that this provided a heightened sensitivity to the data collection and analysis process. Despite the above limitations, the data has strong validity and represent the direct experience of care in two adult acute hospitals.

### Implications for practice and research

This study took place within the context of both hospitals’ Hospice Friendly Hospitals Programmes. Study findings and recommendations now form a key part of the hospital’s systems strategic quality improvement plans for EoLC. A quality improvement plan was devised to address the study findings and recommendations, which are being implemented by each hospital’s end-of-life care committee. A member of the hospital executive management team chairs this committee. System wide initiatives from this plan are discussed at the Quality and Patient Safety Steering Committee whose focus is on quality improvement throughout each hospital. Since completion of the study, implementation of key recommendations has resulted in measurable improvements such as an increase in the number of patients dying in single occupancy rooms and the completion of several projects to enhance the environment for dying patients and their relatives. Other initiatives have included working with colleagues, in the Hospice Friendly Hospitals programme, on the development of a booklet on the provision of information about what to expect when someone is dying [127] and heightened awareness and improvements in communication to bereaved families when there is a Coroners Post Mortem. In addition, EoLC training and education for staff has been revised to improve communication and enhance the provision of spiritual and emotional support informed by bereaved relatives’ recommendations. By collating data and advocating for previously unheard patient’s and relative’s narratives, this research has filled a gap in how acute hospitals can measure EoLC in a meaningful way that leads directly to quality improvements. Further research is required to explore if the issues raised here are reflected in other acute hospitals.

In an Irish context, seeking the views of bereaved relatives should be considered by all hospitals and healthcare settings to ascertain the quality of care at end of life and to support the development of quality EoLC. A recommendation from this study is that this should be conducted at a national level to support benchmarking and EoLC quality improvements, which is already in existence in other jurisdictions [30].

## Conclusion

This paper adds to the body of research ascertaining the quality of EoLC in acute hospitals from the perspective of bereaved relatives. Overall, respondents rated the quality of care as high; however, areas of care which could be improved were also identified. Our findings can advance acute hospital care providers’, policy makers’ and educationalists’ understanding of bereaved relatives experiences and priorities for service improvement.

This research has provided a powerful snapshot in time into what works well and what should be improved to enhance care at end of life. Furthermore, it indicates that a systems-wide approach needs to be undertaken to enhance the experience of care for all dying persons and their relatives.

## Data Availability

The dataset generated and analysed for this study is not publicly available as the conditions of consent from the local ethics review committee of the institutions involved and the formal consent process did not include sharing the dataset.

## Abbreviations

ACP: Advance Care Planning
EoLC: End-of-Life Care
